# Advances in the development of a vaccine for the prevention of human leptospirosis: a review

**DOI:** 10.1590/S1678-9946202668055

**Published:** 2026-08-21

**Authors:** Michele Silva de Jesus, Maria Edilene Martins de Almeida, Luís André Morais Mariúba

**Affiliations:** 1 Fundação Oswaldo Cruz-Fiocruz Amazônia, Instituto Leônidas e Maria Deane, Manaus, Amazonas, Brazil; 2 Fundação Oswaldo Cruz-Fiocruz Amazônia, Instituto Leônidas e Maria Deane, Programa de Pós-Graduação em Biologia da Interação Patógeno-Hospedeiro, Manaus, Amazonas, Brazil; 3 Universidade do Estado do Amazonas, Programa de Pós-Graduação em Ciências Aplicadas à Hematologia, Manaus, Amazona, Brazil; 4 Universidade Federal do Amazonas, Programa de Pós-Graduação em Imunologia Básica e Aplicada, Manaus, Amazonas, Brazil; 5 Universidade Federal do Amazonas, Programa de Pós-Graduação em Biotecnologia, Manaus, Amazonas, Brazil; 6 Fiocruz Rondônia, Instituto Nacional de Ciências e Tecnologia em Biodiversidade, Biotecnologia, Biometeorologia e Toxicologia Aplicadas à Saúde Única, Porto Velho, Rondônia, Brazil. E-mail: andre.mariuba@fiocruz.br

**Keywords:** Leptospirosis, Vaccine, Zoonosis, Antigens

## Abstract

Leptospirosis is a zoonosis with global distribution caused by spirochetes of the genus *Leptospira*. In regions with poor sanitation, leptospirosis is considered a neglected disease, resulting in approximately one million cases and thousands of deaths annually. Transmission occurs mainly through contact with the urine of infected rodents, and clinical manifestations range from mild symptoms to the severe form known as Weil’s disease. Although licensed vaccines are available, they are targeted at specific risk groups. Bacterins are composed of specific serovars, induce a short-term immune response, and may cause adverse reactions. Advances in molecular biology-including sequencing of the *Leptospira* genome-have spurred the development of new vaccination strategies, particularly protein subunit vaccines. These vaccines are based on proteins located in the outer membrane of pathogenic *Leptospira* species. These antigens are expressed in prokaryotic hosts, exhibit high immunogenicity, and, because they are directly exposed to the host’s immune system, are considered promising targets for the development of safer and more effective vaccines. Among the main antigens studied, the most notable are LipL32, LipL41, OmpL1, OmpL37, Loa22, LigA, and LigB. However, the identification of antigens capable of inducing an effective immune response while containing conserved regions across different serovars remains a major challenge in developing a universal vaccine against leptospirosis. This review presents the principal outer membrane proteins of pathogenic *Leptospira* that are considered promising candidates for new vaccine formulations, as well as their role in inducing the immune response in the human host.

## INTRODUCTION

Leptospirosis is a zoonosis with global distribution caused by pathogenic bacteria of the genus *Leptospira*. Approximately one million cases occur annually, resulting in more than 60,000 deaths. Factors such as inadequate sanitation, social inequality, and warm, humid tropical climates contribute to the spread of the disease, particularly during rainy seasons when floods and waterlogging facilitate transmission^1^.

In the transmission cycle, rodents serve as the primary reservoirs and disseminators of *Leptospira*. Owing to their ability to persistently colonize rodent kidneys, the bacteria are frequently excreted into the environment through urine. Humans are considered accidental hosts, acquiring the infection through direct contact with rodent urine or indirectly through contact with contaminated soil or water^2^.

The classification of the genus *Leptospira* has evolved over time. Initially, species were grouped into two categories: saprophytic (*L. biflexa* sensu lato) and pathogenic (*L. interrogans* sensu lato). This classification was later redefined to include saprophytic, intermediate, and pathogenic lineages. With advances in genomic analyses and next-generation sequencing technologies, the genus has been expanded to include 74 species distributed into two major clades, saprophytic (S) and pathogenic (P), which are further subdivided into the subclades S1, S2, P1, and P2, respectively^3^.

This taxonomic diversity is directly associated with the wide range of pathogenic species responsible for infections in humans and animals. Most infections are caused by eight pathogenic species, distributed in 26 serogroups, with more than 300 pathogenic serovars having been identified^3^. In humans, the clinical manifestations of leptospirosis can range from asymptomatic infections to fatal cases caused by renal failure or pulmonary hemorrhage. The severe form of the disease, known as Weil’s disease, is characterized by a triad of symptoms: renal failure, jaundice, and hemorrhage^4^.

Commercially available vaccines for the prevention of leptospirosis, widely used in dogs, cattle, and swine, are formulated from whole, inactivated *Leptospira* cells, known as bacterins. These vaccines protect by triggering a humoral immune response against lipopolysaccharide (LPS), the main antigen located on the bacterium’s outer membrane. However, because LPS is considered T-independent, it does not promote the formation of immunological memory cells, resulting in short-term immunity and consequently requiring annual booster doses. Furthermore, these vaccines provide limited protection across different *Leptospira* serovars, posing a significant challenge for the development of universal vaccines against leptospirosis^5^.

Although bacterins have been approved for human use in countries such as Japan, France, and Cuba, their use is limited to occupational risk groups, individuals living in regions with heavy rainfall, or endemic areas. Despite efforts to immunize certain populations, bacterins have high rates of adverse reactions, are serovar-specific, and induce short-term immunity^6^.

To overcome these limitations, research has focused on the development of protein subunit vaccines. These proteins, located on the outer membrane surface of *Leptospira*, contain conserved regions in most pathogenic serovars, are highly immunogenic, and exhibit few adverse effects, making them promising vaccine candidates for inducing more effective immune responses^7^. Some proteins, such as OmpL37, LipL32, LipL21, LigA, LigB, and LcpA, have been extensively investigated as potential vaccine candidates for the prevention of leptospirosis^8^.

Despite their favorable surface localization and immunogenic properties, their protective efficacy varies across different experimental models. Moreover, limitations such as restricted cross-protection among serovars and antigenic heterogeneity among pathogenic strains remain significant challenges. In this review, we discuss the main outer membrane proteins of *Leptospira* as potential vaccine targets, as well as their role in the activation of the immune response in human hosts. The survey of indexed publications was conducted using the PubMed, Google Scholar, SciELO, and CAPES Journals databases. Only publications in English were included, primarily from 1991 to 2026. The keywords used in the search were lipoprotein, leptospirosis, vaccine, vaccine antigens, and chimeric protein.

## Pathogenesis and immune response in leptospirosis

Infection begins when the bacteria enter through cuts or abrasions in the skin or through the mucous membranes of the oral cavity and conjunctiva, spreading through the bloodstream to reach vital organs such as the lungs, liver, and kidneys. Clinical manifestations range from mild to fatal, and the severity of the infection depends on several factors, including the *Leptospira* serovar responsible for the infection, the bacterial load, and the immune status of the infected individual^9^.

Virulence factors such as hemolysins, flagella, lipopolysaccharides (LPS), and outer membrane proteins play an active role in the interaction with the host’s immune system. Periplasmic flagella, for example, located at each end of the bacterium, are responsible for motility and, through rotational and translational movements, enable penetration into host tissues^10^.

Studies using gene inactivation of the FlaA and FliY proteins, structural components of *Leptospira* flagella, have demonstrated a reduction in virulence in infected animals. These findings indicate that, in addition to their role in bacterial motility, flagella play an essential role in the activation of pathogenic mechanisms^11^.

During infection, the primary defense mechanism is the humoral immune response. The immune system primarily produces antibodies against LPS, a complex molecule located on the outer membrane of *Leptospira*. LPS plays a crucial role in virulence and in the antigenic diversity among serovars. Because it is a highly variable and atypical structure, LPS is recognized differently by pattern recognition receptors (PRRs), rendering the innate immune response ineffective^9^.

Typically, the LPS of Gram-negative bacteria interacts with TLR4 (Toll-like receptor 4), triggering a cytokine- and chemokine-mediated inflammatory response. However, *Leptospira* LPS, being less endotoxic, is recognized by TLR2 receptors in the presence of CD14. Studies have shown that leptospiral LPS is not recognized by TLR4 in human cells, unlike in mouse cells, where LPS is recognized by both TLR2 and TLR4 pathways, resulting in a more robust and effective immune response^12^.

This difference in TLR2 and TLR4 recognition patterns of leptospiral LPS is attributed to structural alterations in lipid A, mediated by phosphate methylation, which is observed only in *Leptospira* LPS and not in LPS from other Gram-negative bacteria. This structural modification is essential for preventing the activation of innate immune cells, serving as a bacterial strategy to evade the host immune system^13^.

Surface lipoproteins are also recognized during the innate immune response by TLR2 receptors. These molecules actively participate in adhesion and cellular invasion, which are essential steps for establishing infection. Thus, both LPS and lipoproteins are considered key elements in the initial immune activation during infection^14^.

Yang *et al.* ^15^ showed the involvement of LipL32 in the infection process of renal tubules by evaluating TLR2 expression in mice infected with pathogenic *Leptospira*. The results confirmed that TLR2 receptors recognize the protein, activate macrophages, and initiate the inflammatory process in the kidneys.

LipL41 and LipL21 also play important roles during infection by interacting with host extracellular matrix (ECM) components such as collagen and fibrinogen. Similarly, Lig proteins (LigA, LigB, and LigC) promote adhesion to host tissues and contribute to host-pathogen interactions. These ECM interactions facilitate *Leptospira* colonization and dissemination^16^.

## Bacterin vaccines

Following the first recorded cases of leptospirosis in Japan, the disease began to spread to other regions of the world. In response, vaccination strategies were developed to reduce the number of infections. Experimental trials using whole, inactivated *Leptospira* cells, known as bacterins, were subsequently initiated^17^.

The first study to evaluate the efficacy of vaccines against leptospirosis was conducted in Japan in 1916, when researchers tested whole *Leptospira* inactivated with phenol in guinea pigs. The results demonstrated the induction of active protective immunity. Based on these findings, Japanese miners were immunized, resulting in a significant reduction in the disease^18^. Noguchi^19^ conducted studies with inactivated *Leptospira* by administering three doses in guinea pigs and demonstrated the induction of active immunity lasting up to eight weeks.

Bacterins-commercial vaccines composed of whole *Leptospira* cells inactivated by heat or formalin-are widely used in veterinary medicine and are licensed for administration in dogs, horses, cattle, and swine. However, their use in humans has not been widely approved and is restricted to certain high-risk groups in some countries^6^.

Although they have been studied for over 100 years, bacterins still have limitations, such as high rates of adverse reactions, serovar-specific immunity, and short-term efficacy. They are formulated according to the epidemiological profile of each country, meaning that different serovars may circulate differently depending on the country or geographic region^20^.

In general, bacterins induce a serovar-specific immune response directed against the LPS present in the vaccine formulation, and the response does not provide cross-protection against other serovars. Furthermore, LPS is considered T-independent, which prevents the production of immunological memory cells. As a result, these vaccines induce short-term immunity, requiring booster doses to maintain protective immunity^21^.

In some countries, such as France, China, Cuba, and Japan, bacterins have been licensed for human immunization against leptospirosis. However, their use is limited to high-risk groups such as sewage workers, livestock farmers, agricultural workers, and veterinarians. Additionally, in climate-related emergencies, such as periods of heavy rainfall followed by flooding, individuals exposed to environments contaminated with *Leptospira* may also be immunized^22^ (Table 1).

**Table 1:** Commercially available and licensed bacterin vaccines against leptospirosis. Inactivated whole-cell formulations, their manufacturers, and the *Leptospira* serovars included in each formulation. These vaccines primarily induce serovar-specific humoral immune responses, with limited cross-protection and the need for booster doses. Source: Xu and Ye^22^.

Vaccine type	Manufacturer (country)	***Leptospira* serovars included**
Inactivated trivalent vaccine	Finlay Institute (Cuba)	Icterohaemorrhagiae, Canicola, Pomona
Inactivated monovalent vaccine	Pasteur Institute (France)	Icterohaemorrhagiae (serogroup Icterohaemorrhagiae)
Inactivated multivalent vaccine	Wuhan Institute of Biologicals (China)	Lai, Grippotyphosa (serovar Linhai), Autumnalis, Canicola, Pomona, Australis, Hebdomadis

In France, the Spirolept^®^ vaccine, developed by the Pasteur Institute, has been in use since 1979. It is composed of the inactivated *L. interrogans* serogroup *Icterohaemorrhagiae* strain, which, according to data from the French Leptospirosis Reference Center, is considered the main circulating serogroup in the country. However, the use of Spirolept^®^ is restricted to high-risk groups, primarily due to the side effects associated with the vaccine^23^.

Between 1991 and 1995, Cuba experienced an increase in morbidity rates from leptospirosis. For this reason, researchers began developing a vaccine, including controlled clinical trials to evaluate its efficacy. In 1998, Vax-SPIRAL^®^, composed of the serovars *L. Icterohaemorrhagiae*, *L. Canicola*, and *L. Pomona*, was licensed and incorporated into Cuba’s Leptospirosis Prevention and Control Program^24^.

In China, since 2007, a multivalent vaccine composed of serogroups *Icterohaemorrhagiae* serovar Lai, *Grippotyphosa* serovar Linhai, *Autumnalis* serovar Autumnalis, *Canicola* serovar Canicola, *Pomona* serovar Pomona, *Australis* serovar Australis, and *Hebdomadis* serovar Hebdomadis has been licensed and incorporated into the country’s Expanded Immunization Program. Like other bacterins, its efficacy is limited to the serovars included in the formulation, and was based on local epidemiological studies, though it covers more than 80% of the serovars circulating in the country^22^.

## Recombinant protein subunit vaccines

In 2003, the genome of *L. interrogans* serovar Copenhageni was sequenced in Brazil. This sequencing helped identify genes related to biosynthetic pathways, toxin production, and lipoproteins, contributing significantly to the understanding of pathogenesis and advancing research focused on identifying potential vaccine candidates against leptospirosis^25^.

In this context, researchers have turned to new strategies, such as the development of protein subunit vaccines, to induce more robust and effective immune responses. These vaccines use specific, highly immunogenic proteins, such as outer membrane proteins^6^. The main recombinant proteins include LipL32, LipL21, OmpL1, OmpL37, LigA, LigB, and Loa22.

**Figure 1:**
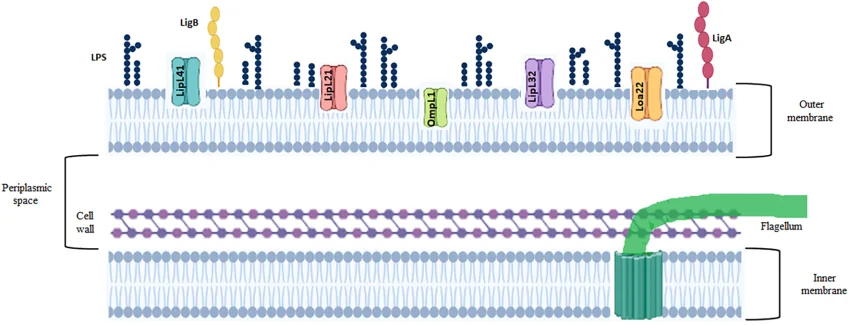
Schematic representation of the cellular structure of *Leptospira*, highlighting major surface-exposed proteins involved in adhesion, immune recognition, and pathogenesis. The outer membrane contains lipopolysaccharides (LPS) and surface-exposed proteins, including LigA, LigB, LipL41, LipL32, and LipL21, as well as the porin OmpL1. The inner membrane is closely associated with the peptidoglycan cell wall located within the periplasmic space. The flagellar structure, responsible for *Leptospira* motility, is embedded in the inner membrane and located within the periplasmic space.

These proteins are associated with bacterial adhesion, pathogen-host interactions, and virulence mechanisms and, due to their surface localization, are exposed to the host immune system. In addition, many of these proteins contain conserved regions among different serovars, a characteristic that is important for the development of a universal vaccine with broader protective capacity. Despite their high immunogenicity, vaccine efficacy varies due to factors such as differences in expression during infection, antigenic heterogeneity among strains, and variation in immune responses across experimental models^26^ (Figure 1).

In this regard, functional studies have contributed to a better understanding of the biological role of these antigens in leptospiral pathogenesis. Fernandes *et al.* ^27^, using CRISPR-based gene editing, investigated the functional role of the proteins LipL21, LipL32, LipL41, and OmpL1 from *L. interrogans* serovar Copenhageni. Silencing of the *lipL32* gene resulted in increased virulence, as evidenced by the earlier onset and greater severity of clinical symptoms in infected animals. In contrast, silencing the *lipL41* gene caused a slight attenuation of virulence, reflected by increased survival rates in lethal challenge assays. The inactivation of OmpL1 compromised bacterial viability, with no viable colonies observed, whereas disruption of the *lipL21* gene resulted in the inability to establish acute infection.

These results highlight the critical role of these proteins in leptospiral pathogenesis and reinforce their relevance as strategic targets for the development of subunit vaccines and chimeric formulations. The functional characterization of these antigens therefore contributes to the rational selection of vaccine candidates with greater protective potential.

## Outer membrane proteins of Leptospira as vaccine candidates

Outer membrane proteins (OMPs) have been extensively investigated as vaccine antigens against leptospirosis. Haake *et al*.^28^ evaluated the immunoprotective capacity of the porin OmpL1 and the lipoprotein LipL41 in hamsters. For this purpose, the gene sequences encoding these proteins were cloned into expression vectors and produced in *Escherichia coli*. Active immunization, followed by challenge with *L. kirschneri* serovar Grippotyphosa, resulted in a survival rate of 71% after 28 days. This study was among the first to demonstrate the potential of protein subunit vaccines to induce protection against leptospirosis. However, the partial protection observed indicates that challenges related to protective efficacy still persist. Thus, these findings contributed to further investigations on OMPs as promising vaccine candidates.

Oliveira *et al.* ^29^ evaluated the immunoprotective potential and the induction of both humoral and cellular immune responses of the OmpL37 protein, derived from *L. interrogans* serovar Copenhageni, using three distinct approaches: protein subunit vaccine, DNA vaccine, and a prime-boost strategy in hamsters subjected to lethal challenge. The protein subunit vaccine induced significant levels of IgG antibodies, whereas the prime-boost strategy elicited a humoral response only after the second dose. In contrast, the DNA vaccine did not induce a detectable humoral response. However, none of the vaccination strategies conferred effective protection against infection, highlighting limitations in the protective potential of the OmpL37 protein and emphasizing the need to identify new functional vaccine targets.

Schuler *et al.* ^30^ investigated the Loa22 protein (*Leptospira* OmpA-like protein), an important virulence factor, as a vaccine antigen, evaluating its potential in chimeric vaccine formulations. Amino acid sequences were retrieved from the NCBI database, aligned using ClustalW, and phylogenetically analyzed with the MEGA X software, whereas B-cell epitopes were predicted using the BepiPred-2.0 tool. The analyses identified epitopes with potential bactericidal activity, and immunization with vaccines based on these epitopes induced high IgG titers in mice. However, the level of immunoprotection ranged from 50% to 80%, indicating that Loa22 has limitations as a standalone vaccine candidate, despite being immunogenic. Therefore, the authors suggest combining epitopes from different proteins as a strategy to enhance immunological efficacy and achieve more robust protection against leptospirosis.

Altogether, the highlighted studies reveal the limitations of approaches based on single antigens and reinforce the need to investigate alternative vaccine strategies and combinations of multiple antigens. Other *Leptospira* proteins, including those of the LipL family, have also been extensively investigated as potential vaccine candidates.

## Lipoprotein of Leptospira (LipL)

Most lipoproteins of pathogenic *Leptospira* are located on the bacterial surface, display conserved regions among different serovars, and directly interact with components of the host extracellular matrix. These proteins play an important role in pathogenesis and are associated with bacterial adhesion, tissue colonization, and modulation of the host immune response, in addition to exhibiting the potential to induce immunological responses. Among these proteins, LipL32 is the most abundant outer membrane lipoprotein expressed by pathogenic *Leptospira* species and has been widely investigated as a potential vaccine candidate^31^.

LipL32, also known as hemolysis-associated protein 1 (Hap-1), is considered the most abundant lipoprotein in the outer membrane of pathogenic *Leptospira*. This protein has been extensively investigated as a vaccine target in the development of recombinant vaccines due to its high immunogenicity, the presence of conserved regions among different serovars, its expression during infection, and its exclusive occurrence in pathogenic species. In addition, anti-LipL32 antibodies have been detected in approximately 95% of sera from infected patients. However, despite its high immunogenicity and widespread expression, experimental studies have reported variable results regarding its protective efficacy, indicating limitations when used as a single vaccine candidate^32^.

Seixas *et al.* ^33^ ^)^ cloned the *lipL32* gene into four expression vectors (pUS973/*lipL32*, pUS974/*lipL32*, pUS977/*lipL32*, and pUS2000/*lipL32*), which were introduced into recombinant *Mycobacterium bovis* BCG by electroporation. Groups of hamsters were immunized with the different constructs and subsequently challenged with a lethal dose of *L. interrogans* serovar Copenhageni. The vaccines rBCG/pUS973/*lipL32* and rBCG/pUS974/*lipL32* did not induce detectable antibody levels and showed low protection rates. In contrast, the vaccines rBCG/pUS977/*lipL32* and rBCG/pUS2000/*lipL32* induced high antibody levels in immunized animals and demonstrated greater protective potential. However, the variation in the immune response observed among the different constructs highlights the influence of the expression system on vaccine efficacy and reinforces the need to optimize antigen delivery strategies.

In a similar study, Grassmann *et al.* ^34^ evaluated the LipL32 protein fused to the heat-labile toxin B subunit of *E. coli* (LTB) in hamster challenge experiments using a dose five times higher than the lethal dose. Immunization with the LTB-LipL32 formulation resulted in a survival rate of 60% compared to the control group, representing one of the first reports of partial protection against lethal leptospirosis. Although these findings demonstrated increased protection, the partial efficacy observed suggests that combinatorial strategies or the use of additional adjuvants may be required to achieve more robust protection.

Subsequent studies have continued to investigate the immunogenicity and protective efficacy of LipL32 using different vaccine formulations and experimental models. Seixas *et al*.^35^, aiming to evaluate the immunogenicity of LipL32, employed different vaccination strategies, including a DNA vaccine based on the *lipL32* gene cloned into the pTarget vector, recombinant BCG constructs containing the *lipL32* gene (pUS973, pUS974, and pUS977), and a protein subunit vaccine consisting of recombinant LipL32 produced in *Escherichia coli* following expression of the *lipL32* gene cloned into the pAE vector. In mice divided into eight experimental groups, all formulations induced high levels of anti-LipL32 antibodies. In the group that received a booster dose of rLipL32, high antibody titers were observed 42 days after the first immunization and remained elevated until the end of the experiment (160 days). Moreover, antibody titers induced by the rBCG vaccine continued to increase throughout the experimental period. Indirect immunofluorescence analysis confirmed LipL32 as a surface antigen, demonstrating that the antibodies recognized the intact protein on the membrane of *L. interrogans*, reinforcing its potential as a vaccine candidate. Despite the high immunogenicity observed, the correlation between the humoral response and effective protection against infection still requires further investigation.

Recombinant vaccines using outer membrane proteins continue to be extensively investigated due to the direct involvement of these proteins in activating the host immune response and in the pathogenesis process. Besides LipL32, other outer membrane proteins such as LipL21, LipL41, and LipL36 are also considered potential vaccine targets^36^.

LipL41 was the first lipoprotein identified in *Leptospira kirschneri* strains through solubilization with Triton X-114, while LipL21 was isolated from *Leptospira interrogans* serovar Lai and characterized as a lipoprotein in assays using palmitic acid. LipL21 is the second most abundant protein in the outer membrane and contains conserved regions among different *Leptospira* serovars. These structural and functional characteristics have contributed to the growing interest in investigating these proteins as potential vaccine candidates^28^.

Takahashi *et al*.^16^ ^)^ evaluated the interactions of the proteins LipL21 and LipL41 with extracellular matrix (ECM) components, plasma proteins, glycosaminoglycans (GAGs), cellular receptors, and other plasma components. The study demonstrated a broad binding spectrum of these proteins to ECM molecules, characterizing them as multifunctional adhesins involved in the early stages of infection, in addition to exhibiting high immunogenic potential. These findings reinforce the relevance of these proteins as promising targets for the development of new vaccine formulations as well as for diagnostic methods. However, additional studies are required to determine whether these properties translate into effective protection against infection in experimental models. Outer membrane lipoproteins therefore play a central role in the pathogenesis of leptospirosis and remain important targets in the search for more effective vaccines.

## Leptospiral immunoglobulin-like (Lig) proteins

In 2001, a new family of outer membrane proteins, termed leptospiral immunoglobulin-like (Lig) proteins, was described, comprising LigA, LigB, and LigC. However, in some *Leptospira* species, LigC is considered a pseudogene and does not play an essential role in virulence^37^.

These proteins were identified in the screening of expression libraries of *L. interrogans* and *L. kirschneri* using sera from patients with leptospirosis. The sequences corresponding to Lig proteins were found exclusively in pathogenic species, and their localization on the bacterial surface was confirmed by high-pressure freezing techniques combined with immunocytochemical electron microscopy. Moreover, immunoblot assays using patient sera demonstrated that Lig proteins are among the major antigens recognized during the acute phase of infection^38^.

Currently, the proteins LigA and LigB are among the most extensively investigated surface antigens in studies toward the development of vaccines against leptospirosis. These proteins contain domains belonging to the bacterial immunoglobulin-like (Big) family and share structural similarities with known virulence factors, such as invasin from *Yersinia pseudotuberculosis* and intimin from *E. coli*. They are capable of interacting with several components of the extracellular matrix, playing an important role in bacterial adhesion and colonization processes during *Leptospira* infection. These structural and functional characteristics have contributed to the growing interest in investigating these proteins as potential vaccine candidates^39^.

Although proteins of the Lig family have shown promising results in inducing protective immunity during the acute phase of leptospirosis, the LigA protein does not contain conserved regions across all pathogenic species, which limits its ability to induce cross-protection. Studies conducted by Bulach *et al.* ^40^ and Choy *et al*.^41^ demonstrated the absence of sequences encoding this protein in species such as *L. borgpetersenii* serovar Hardjo and *L. interrogans* serovar Lai. This limitation represents an important challenge for the use of LigA as a vaccine antigen for leptospirosis prevention, highlighting the importance of identifying more conserved antigens or developing multicomponent vaccine strategies to broaden protection against different pathogenic *Leptospira* species.

In experimental studies using an acute leptospirosis hamster model, the ability of LigA and LigB proteins to induce renal sterilizing immunity and protection against lethal *Leptospira* infections was evaluated. For this purpose, vaccines formulated with these recombinant proteins were tested individually and in combination. Immunization with the Lig proteins induced high antibody titers, eliciting a strong humoral response. Immunization with LigA alone was effective in protecting against lethality but did not prevent renal infection. In animals immunized with LigB alone, only three of the original eight hamsters survived the challenge test. The combined LigA and LigB formulation was not effective in protecting the kidneys against bacterial colonization, nor in reducing the bacterial load in the renal tubules^39^.

These findings indicate that, despite their high immunogenicity, Lig proteins present limitations in inducing sterilizing immunity, highlighting the difficulty of completely preventing renal colonization. Although Lig proteins represent important vaccine targets, their limitations underscore the need for combinatorial strategies or the identification of new, more conserved antigens.

## Multi-epitope vaccines: recombinant chimeric proteins

Advances in reverse vaccinology and comparative genomics have made it possible to identify conserved regions across pathogenic *Leptospira* species, enabling the development of multi-epitope vaccines. These approaches allow the selection of B- and T-cell epitopes with the potential to enhance cross-protection and overcome the limitations associated with serovar-specific immunity. Multi-epitope vaccine strategies have thus emerged as a promising approach for the development of broad-spectrum vaccines against leptospirosis^42^.

Based on this approach, recent studies have focused on the development of chimeric proteins to induce more effective immune responses against leptospirosis while overcoming limitations observed with the use of single antigens, such as partial protection and antigenic variability. These constructs are composed of immunogenic epitopes selected from outer membrane proteins of pathogenic *Leptospira* species. They stimulate specific immune responses, broaden protection against different serovars, and offer a promising strategy for the development of broad-spectrum vaccines^43^.

Several studies have evaluated different chimeric constructs based on conserved epitopes from *Leptospira* proteins, and their results have identified promising vaccine candidates when tested in experimental models. Lin *et al.* ^8^, while evaluating immunogenicity against *L. interrogans* and cross-protection among different serovars, constructed a chimeric protein containing B- and T-cell epitopes selected from the proteins OmpL1, LipL21, and LipL32. Immunization induced a strong immune response in guinea pigs, resulting in high antibody production, a higher survival rate (80%) compared to the control group, reduced renal colonization, and decreased urinary shedding of *Leptospira*. In addition, in microscopic agglutination tests, the chimera exhibited cross-reactivity against serogroups of reference *Leptospira* strains isolated in China.

Collectively, these data indicate that multi-epitope-based strategies may enhance protective responses and overcome limitations observed in vaccines based on single antigens, representing a promising approach for the development of leptospirosis vaccines.

Similar results have been reported for other chimeric constructs based on conserved epitopes. Fernandes *et al*.^44^, aiming to develop a vaccine capable of inducing cross-protective immunity against different leptospiral serovars, constructed a chimeric protein containing B- and T-cell epitopes derived from the proteins LigA, Mce, Lsa45, OmpL1, and LipL41. The gene encoding this chimera was cloned into the pAE vector and expressed in *E. coli*. Recognition of the chimeric protein by antibodies present in sera from infected patients confirmed the immunogenic potential of the selected epitopes. In challenge experiments, hamsters immunized with the chimera developed high IgG antibody titers. However, the observed protection rate was limited to 50%.

The authors attributed this low protection either to non-protective epitopes or to masking of antigenic regions on the LigA protein-a key vaccine candidate-thereby compromising the immune response. These findings indicate that, although multi-epitope strategies show promising potential, the appropriate selection of protective epitopes and preservation of antigenic structure are critical factors for the development of effective vaccines against leptospirosis.

Oliveira *et al.* ^45^ evaluated immunoprotection and the ability to induce sterilizing immunity in hamsters using two chimeric constructs encoding epitopes derived from LipL32, LigANI, LigBrep, and LemA. The constructs were expressed in Mycobacterium bovis Bacillus Calmette-Guérin (BCG), which was used as a delivery system, and tested under the control of different promoters (pAN, Hsp60, 18 kDa, and Ag85B). Chimera 1 (LipL32, LigANI, and LemA) showed protection rates ranging from 80% to 100% in lethal challenge experiments, regardless of the promoter used. Chimera 2 (LigANI and LigBrep) conferred satisfactory protection only when expressed under the control of the pAN promoter. Both constructs induced a significant humoral response, with high IgG antibody titers after a single vaccine dose; however, they were not able to induce sterilizing immunity in the kidneys.

These results indicate that although chimeric vaccines may provide high protection against lethal challenge, preventing renal colonization remains a challenge, highlighting the difficulty of inducing sterilizing immunity.

In studies by Tapajóz *et al.* ^46^, a chimeric protein was also constructed using immunodominant epitopes from the lipoproteins LIC12287, LIC11711, and LIC13259, identified via conserved region analysis and B- and T-cell epitope mapping. Hamsters immunized with the chimera demonstrated a strong humoral response, with high IgG production detectable up to 28 days after the second dose, confirming a specific response to the chimera’s epitopes. However, in challenge assays with a virulent *L. interrogans* strain, effective protection was not observed. Although the chimera induced a strong humoral response, the authors emphasized the need for new epitope combinations capable of eliciting effective protection against leptospirosis.

These results indicate that high immunogenicity does not necessarily indicate effective protection, highlighting challenges in selecting epitopes with real protective potential. In summary, studies involving chimeric proteins and multi-epitope vaccines have demonstrated promising results in inducing robust immune responses and protection against lethality. However, challenges related to the induction of sterilizing immunity and the selection of highly protective epitopes still limit their application as a universal vaccine strategy against leptospirosis.

Moreover, advances in reverse vaccinology and comparative genomics have enabled the identification of conserved regions across pathogenic *Leptospira* species, contributing to the rational design of multi-epitope vaccines. These approaches allow the selection of B- and T-cell epitopes with the potential to enhance cross-protection and overcome the limitations associated with serovar-specific immunity. Therefore, the integration of reverse vaccinology with multi-epitope strategies represents a promising approach for the development of broadly protective vaccines against leptospirosis.

## DNA vaccines

The development of new vaccine platforms has been widely explored in the search for a universal vaccine capable of inducing more effective, protective, and safe immune responses against the various serovars of *Leptospira*. DNA vaccines are considered one of the simplest forms of vaccine development. These vaccines are composed of plasmids that contain genetic sequences encoding one or more vaccine candidate proteins. Their functional structure includes regulatory elements required for the transcription and translation of these sequences within host cells^47^ (Table 2).

**Table 2:** Vaccine strategies against leptospirosis and their main characteristics. Comparison of different vaccine platforms, including antigenic composition, animal models, protective efficacy, immune responses, and main limitations. These approaches range from conventional bacterins to emerging platforms, such as multi-epitope and mRNA-based vaccines, highlighting the challenges in achieving broad and long-lasting protective immunity.

Vaccine type	Antigen (s) used	Animal model	Protective efficacy	Immune response	Main limitations	Articles
Inactivated whole-cell vaccines (bacterins)	Whole *Leptospira* cells (LPS-based immunity)	Guinea pigs, hamsters	Serovar-specific, short-term protection	Predominantly humoral	Limited cross-protection, frequent boosters, reactogenicity	Adler^20^; Haake and Levett^17^
Recombinant protein vaccines	LipL21, LipL32, LipL41, LigA, LigB, OmpL1, Loa22	Hamsters, mice	Partial protection	Humoral and cellular immune responses	Variable efficacy, limited cross-protection, antigenic variability	Dellagostin *et al*.^6^, Murray^32^, Cullen *et al*.^36^, Evangelista *et al*.^39^
DNA vaccines	*ligA*, *ligB*, *lipL32* (genes)	Mice, hamsters	Partial protection	Humoral and cellular immune responses	Low immunogenicity in humans, limited clinical translation	Silveira *et al*.^48^, Forster *et al*.^49^, Wang *et al*.^60^
Multi-epitope (chimeric) vaccines	Conserved immunogenic epitopes	Mice, guinea pigs	Partial protection	Targeted humoral and cellular immune responses	Limited *in vivo* validation, inconsistent protection	Lin *et al*.^8^, Oliveira *et al*.^29^
mRNA vaccines	LigA, LigB	Mice, hamsters	Partial protection	Humoral and cellular immune responses	Low protective efficacy; requires further optimization	Techawiwattanaboon *et al*.^59^

DNA vaccines are generally administered via the intramuscular route in host tissue, allowing *in vivo* expression of antigens in cells and consequently the induction of an immune response to the expressed proteins. Compared to traditional vaccines such as bacterins, DNA vaccines offer several advantages: they induce both humoral and cellular immune responses, stimulate the immune system without the risk of microorganism replication, and are inexpensive, facilitating large-scale production^48^.

Forster *et al*.^49^ evaluated the protective potential of a DNA vaccine composed of fragments of the LigA and LigB proteins derived from the *Leptospira interrogans* serovar Canicola strain. The genes were cloned into the pTARGET expression vector, and hamsters were immunized and subsequently challenged with *L. interrogans* serovar Copenhageni. The formulation containing the LigBrep fragment showed a protection rate of 62.5%, in addition to inducing IgG antibody production and sterilizing immunity in 80% of the surviving animals. On the other hand, the other evaluated fractions (LigAni, LigBni, LigBct1, and LigBct2) did not confer significant protection, despite the induction of a humoral response in the case of the LigAni fraction. Overall, the results indicated that, although DNA vaccines can induce a detectable immune response, immunogenicity alone does not guarantee effective immunoprotection, highlighting the need for vaccine strategies that combine multiple antigens or different approaches to increase efficacy against leptospirosis.

To address this need, a vaccine strategy combining a DNA vaccine and a recombinant protein based on the LipL32 and Loa22 antigens was evaluated using chitosan as a delivery system. A plasmid composed of the *lipL32* and *loa22* protein genes was constructed and administered to mice in a heterologous prime-boost (DNA/protein) immunization. The results demonstrated that the combination of the two antigens induced higher levels of specific antibodies compared to formulations containing individual antigens. An increase in the production of cytokines associated with the cellular immune response, such as IFN-γ and IL-2, was also observed. These findings suggest that DNA vaccines incorporating multiple antigens, together with delivery systems such as chitosan, can enhance immunogenicity and therefore represent a promising strategy for developing more effective vaccines against leptospirosis^50^.

In another study, Vijayachari *et al.* ^51^ designed a plasmid DNA construct encoding the LipL45 protein to evaluate whether the vaccine would be able to induce strong anti-LipL45-specific humoral and cellular immune responses. In *in vivo* assays, mice were immunized via the intramuscular route using electroporation, with a significant cellular response observed, along with the production of anti-LipL45-specific antibodies and the induction of a Th1-type immune response, mainly with increased production of the cytokines IL-12 and IFN-γ. Although the results showed the promising immunogenic potential of the plasmid encoding LipL45, the study did not evaluate the vaccine’s ability to confer protection in challenge assays with virulent *Leptospira* strains. Additional studies are necessary to determine its protective efficacy in experimental infection models.

Overall, DNA vaccines developed against leptospirosis have the ability to induce humoral and cellular immune responses in experimental models. However, new strategies including different types of delivery systems, the combination of multiple antigens, and validation in challenge studies are still needed to enhance their efficacy and applicability in the control of human and animal leptospirosis^48^.

## Reverse vaccinology approaches and -omics technologies in the identification of vaccine candidates

With advances in genomic sequencing, new approaches have been adopted to identify vaccine candidates. The concept of reverse vaccinology, proposed by Rappuoli^42^, is based on the analysis of complete pathogen genomes using bioinformatics tools to predict potential antigens, particularly surface proteins, virulence factors, and proteins conserved among pathogenic species. This strategy emerged as a faster and more promising alternative to the conventional approach, which consists of pathogen cultivation, isolation, and subsequent experimental analyses to identify vaccine targets.

In 2003, using a random sequencing method, Ren *et al.* ^52^ obtained the complete genome sequences of a virulent strain of *L. interrogans* serovar Lai, belonging to the Icterohaemorrhagiae serogroup. Based on their findings, it was shown that the genome was composed of two circular chromosomes, totaling approximately 4,768 predicted genes. About 41% of the coding sequences show no significant similarity to proteins from other organisms, suggesting the presence of unique genetic elements and potential new vaccine targets. In addition, genomic analysis led to the identification of genes involved in motility, outer membrane proteins, lipopolysaccharide (LPS) biosynthesis, and virulence factors.

Subsequently, through comparative genomic analyses with serovar Lai, the genome sequence of the *L. interrogans* serovar Copenhageni strain was described, and new genomic features were identified. Among the main findings are genes associated with transport systems, regulation, and biosynthetic pathways, as well as genes related to lipoproteins and proteins exposed on the outer membrane. These genomic sequences helped to expand the understanding of the molecular mechanisms of leptospiral pathogenesis and broadened the genomic landscape of the genus, providing a stronger basis for the application of reverse vaccinology and enabling the identification of candidates for broad-spectrum vaccines^53^.

Using a bioinformatics approach based on reverse and structural vaccinology, Grassmann *et al*.^54^ ^)^ sought to identify new vaccine candidates from the L1-130 genome of the Copenhageni serovar strain of *L. interrogans*. In this study, 26 targets with vaccine potential were identified, directly involved in the functioning of various biological pathways such as pathogenesis, iron and vitamin transport, outer membrane proteins (OMPs), and LPS assembly. Although this strategy represents a rational and systematic approach for the selection of vaccine antigens, the authors themselves acknowledged that *in silico* prediction alone does not guarantee effective immunoprotection. Thus, the bioinformatic identification of vaccine candidates and their experimental validation remain a central challenge in the development of vaccines against leptospirosis.

With the aim of applying reverse vaccinology associated with cell surface immunoprecipitation (CSIP), Maia *et al*.^55^ selected 22 vaccine constructs derived from protein fragments of 33 β-barrel outer membrane proteins (βb-OMPs) from the *L. interrogans* serovar Copenhageni L1-130 strain. The recombinant constructs contained regions predicted to be surface-exposed and to harbor MHC-II binding epitopes. Although most formulations demonstrated significant immunogenicity, and four constructs induced complete protection and sterilizing immunity, these results were not reproducible in subsequent evaluations. Thus, despite the methodological advances provided by the integration of genomic, structural, and immunoproteomic tools in the selection of vaccine candidates, achieving consistent protection remains a significant challenge in the development of universal vaccines against leptospirosis.

Reverse vaccinology has also been applied to the *Leptospira borgpetersenii* serovar Hardjo strain. The choice of this serovar is particularly relevant due to its importance in bovine leptospirosis and its economic impact, in addition to representing an occupational risk for farmers and veterinarians. Murray *et al*.^56^ conducted a study that evaluated 238 antigens derived from proteins predicted to be outer membrane proteins, which were organized into 48 antigen pools. Although most protein groups induced a detectable humoral response in the hamster model, none conferred protection against infection.

This study provides an important reference on the selection of leptospiral protein candidates; however, it shows that a humoral response alone is not a reliable marker of protection. It underscores the need for more robust functional criteria and a deeper understanding of the immunological mechanisms underlying protection against leptospirosis.

## Perspectives

Despite advances in the development of vaccine platforms against leptospirosis, several challenges remain, particularly regarding the induction of long-lasting immunity and broad cross-protection against different pathogenic Leptospira serovars. mRNA-based vaccines have emerged as a promising alternative to conventional approaches, as they are non-infectious and non-integrative, reducing the risk of infection or insertional mutagenesis and presenting a favorable safety profile. Additionally, they have the potential for high efficacy and allow rapid, low-cost, large-scale production of vaccines^57^ ^), (^ ^58^.

Recent studies have highlighted the potential of mRNA-based vaccines against leptospirosis. Although these approaches are still in early stages, experimental studies using regions of the LigA and LigB proteins from *Leptospira interrogans* serovar Pomona, encapsulated in lipid nanoparticles, have shown promising results, including rapid and robust humoral responses with high IgG levels in murine models. Complement-mediated functional activity and cellular responses associated with a Th1 profile were also observed. However, protective efficacy was limited, with low survival rates. Nevertheless, a reduction in renal colonization indicated a partial protective effect. These findings suggest that mRNA-based vaccines represent a promising strategy, although further optimization-such as selecting more conserved antigens and improving delivery systems-is needed to enhance protective efficacy^59^.

Other potentially promising platforms have emerged, particularly vaccines based on viral vectors. These platforms offer advantages for controlling pathogens that are difficult to manage with conventional approaches and are among the most effective strategies for inducing robust humoral and cellular immune responses. Key viral vectors include influenza viruses, adenoviruses, and poxviruses, recognized for their efficiency in antigen delivery, high immunogenicity, and potential protective efficacy. These platforms also allow flexibility in routes of administration, including mucosal immunization, and have additional applications supporting the rational use of this technology^60^.

Given the limitations of current leptospirosis vaccines, which provide limited protection and short-lived immunity, viral vectors represent a promising strategy. However, challenges related to safety, pre-existing immunity to the vector, and formulation optimization must still be addressed for effective application. Future efforts should focus on selecting conserved antigens, improving delivery platforms, and conducting robust *in vivo* and clinical studies to advance promising candidates for effective human vaccines.

## CONCLUSION

Currently, bacterins remain the only commercially available vaccines in some countries for the prevention of leptospirosis. However, they present significant limitations, including restricted cross-protection among serovars and the inability to induce long-lasting immunity. The major challenges in vaccine development include the identification of highly conserved antigens, the induction of effective immune responses, and the achievement of both protective and sterilizing immunity.

Although recent research has highlighted the potential of protein subunit vaccines in inducing protective immune responses, most formulations based on a single antigen have shown limited efficacy and restricted induction of sterilizing immunity. Therefore, further studies are needed to identify vaccine candidates with higher immunogenic potential.

Mapping functional epitopes on outer membrane proteins is a promising strategy for eliciting stronger immune responses. In this context, the development of multi-epitope chimeric proteins represents a promising approach to overcome the limitations observed in conventional vaccine formulations.

In addition, emerging vaccine platforms, including mRNA-based vaccines, viral vectors, and advanced delivery systems such as nanoparticles, have opened new perspectives for the development of new vaccines against leptospirosis. These approaches offer significant advantages, including improved antigen presentation and the induction of both humoral and cellular immune responses. However, challenges related to antigen selection, optimization of delivery systems, and validation in translational models remain to be addressed. Future studies should prioritize the detailed characterization of the immune responses induced by these strategies, as well as the evaluation of their immunogenic potential and protective efficacy. The combination of multiple epitopes within a single vaccine formulation may represent a viable strategy for the development of a universal, safe, and effective vaccine for the control of leptospirosis.

## Data Availability

The complete anonymized dataset supporting the findings of this study is included within the article itself.
